# Brain Interleukin-1 Facilitates Learning of a Water Maze Spatial Memory Task in Young Mice

**DOI:** 10.3389/fnbeh.2017.00202

**Published:** 2017-10-23

**Authors:** Takako Takemiya, Kumiko Fumizawa, Kanato Yamagata, Yoichiro Iwakura, Marumi Kawakami

**Affiliations:** ^1^Medical Research Institute, Tokyo Women’s Medical University, Tokyo, Japan; ^2^Synaptic Plasticity Project, Tokyo Metropolitan Institute of Medical Science, Tokyo, Japan; ^3^Center for Experimental Animal Models, Institute for Biomedical Sciences, Tokyo University of Science, Chiba, Japan

**Keywords:** interleukin-1β (IL-1β), interleukin-1α (IL-1α), IL-1β knockout mice (IL-1βko), IL-1 receptor 1 (IL-1r1), IL-1r1 knockout mice (IL-1r1ko), water maze (WM), young and adult mice, spatial memory

## Abstract

The proinflammatory cytokine interleukin-1 (IL-1) is produced by many types of cells, including immune cells in the periphery and glia and neurons in the brain. The type I IL-1 receptor (IL-1r1) is primarily responsible for transmitting the inflammatory effects of IL-1 and mediates several biological functions by binding to either IL-1α or IL-1β. IL-1β activation is associated with hippocampus-dependent memory tasks. Although IL-1β impairs spatial memory under certain pathophysiological conditions, IL-1β may be required for the normal physiological regulation of hippocampal plasticity and memory. In addition, brain IL-1β levels are thought to change in the hippocampus in an age-dependent manner. These findings suggest that IL-1β may have a beneficial, temporary effect on learning and memory in young mice, but the matter remains unclear. Therefore, we hypothesized that hippocampal IL-1β has a beneficial effect on spatial learning and memory in young mice via IL-1r1, which is diminished in adults. We investigated the performance of young (3-month-old) and adult (6-month-old) wild-type mice, IL-1β knockout mice (IL-1βko) and IL-1r1 knockout mice (IL-1r1ko) in learning a spatial memory task with a fixed platform in a water maze (WM) and measured the levels of IL-1β and IL-1α in the hippocampus and cortex of adult and young mice by using homogeneous time-resolved fluorescence (HTRF). Learning was significantly impaired in the training trials of the WM spatial memory task in young IL-1βko and IL-1r1ko mice but not in adult IL-1βko and IL-1r1ko mice. Moreover, young IL-1r1ko mice but not IL-1βko mice showed an impairment in long-term memory extinction, suggesting that IL-1α might facilitate memory extinction. In this study, the cytokine assay using HTRF did not indicate a higher expression of hippocampal IL-1 in young mice but cortical IL-1β and IL-1α were significantly increased in adult mice. We need to investigate the role of cortical IL-1 and the local IL-1 expression in the hippocampal neurons in the future.

## Introduction

The proinflammatory cytokine interleukin-1 (IL-1) is produced by many types of cells, including immune cells in the periphery and glia and neurons in the brain (Dinarello, 1996). Interleukin-1 receptor (IL-1r) is a cytokine receptor that binds IL-1. The type I receptor (IL-1r1) is primarily responsible for transmitting the inflammatory effects of IL-1, while the type II receptor may act as a suppressor of IL-1 activity by competing for IL-1 binding. IL-1r1 mediates several biological functions by binding to either IL-1α or IL-1β (Labow et al., 1997). An IL-1 receptor antagonist (IL-1ra) blocks the effects of IL-1 (Dinarello, 1996).

Interleukin-1β activation is associated with hippocampus-dependent memory tasks (Depino et al., 2004; Labrousse et al., 2009) and long-term potentiation (LTP; Schneider et al., 1998; Balschun et al., 2003). Intracerebral injection or microinjection of lipopolysaccharide (LPS) into the hippocampus increases hippocampal IL-1β levels, particularly in the CA1 subregion (Tanaka et al., 2006; Wang et al., 2013). Peripheral stimulation with LPS also increases IL-1β levels in the CA1 hippocampus (Bilbo et al., 2008; Richwine et al., 2008). Therefore, many researchers have investigated the effects of IL-1β expressed in the hippocampal CA1 subregion on learning and memory.

As shown in previous studies, IL-1β impairs learning of a spatial memory task under certain pathophysiological conditions, such as acute inflammation caused by IL-1β injection (Oitzl et al., 1993; Gibertini et al., 1995; Rachal Pugh et al., 2001; Scholz et al., 2016) or IL-1β overexpression (Moore et al., 2009; Hein et al., 2010). These effects were studied using the water maze (WM). IL-1β also inhibits LTP in hippocampal slices (Cunningham et al., 1996; Ross et al., 2003; Scholz et al., 2016). Therefore, IL-1β has inhibitory effects on hippocampus-dependent memory processes.

Recent evidence has suggested that IL-1 may be required for the normal physiological regulation of hippocampal plasticity and the learning process. IL-1ra administration impairs memory formation in the WM (Yirmiya et al., 2002) and fear conditioning (Yirmiya et al., 2002; Goshen et al., 2007) and impairs hippocampal LTP (Schneider et al., 1998), whereas relatively low intracerebroventricular (ICV) doses of IL-1β improve learning of a spatial memory task (Yirmiya et al., 2002; Song et al., 2003; Goshen et al., 2007). In addition, learning a spatial memory task is impaired in mice with a targeted deletion of the IL-1r1 [IL-1r1 knockout mice (IL-1r1ko)], and LTP is absent in these mice in both *in vivo* and *in vitro* experiments (Avital et al., 2003). Another study has demonstrated that mice with transgenic (TG) overexpression of IL-1ra display impairments in spatial memory and contextual memory (Goshen et al., 2007) and show a low rate of successful trials in a T-maze test (Spulber et al., 2009).

Interleukin-1β is also known to have a dose-dependent effect on fear memory. Although low-dose ICV injection of IL-1β (1 ng IL-1β in 2–4-month-old mice or 15 ng IL-1β in young rats weighing 250–280 g) facilitates fear memory (Song et al., 2003; Goshen et al., 2007), high-dose IL-1β (10 ng IL-1β in 2–4-month-old mice) impairs memory performance in young animals (Goshen et al., 2007). In contrast, the effect of low-dose IL-1β (10 ng) is unclear in adult rats; it either facilitates (Yirmiya et al., 2002) or impairs contextual fear conditioning (Rachal Pugh et al., 2001). In addition, Yirmiya et al. (2002) has shown that low-dose IL-1β has no effect on a spatial memory task using a WM in adult rats (6–8 months old). Moreover, 2–4-month-old IL-1r1ko or IL-1ra TG mice showed an impairment in spatial memory performance in a WM (Avital et al., 2003; Goshen et al., 2007); however, the age-dependent effect of a physiological dose of IL-1β on performance in a spatial memory task is unknown. Studies have compared hippocampal IL-1β expression between young and aged mice (1-month and 12-month-old mice, 3-month and 24-month-old mice) (Badowska-Szalewska et al., 2013, 2014) or between adult and aged mice (5–6-month and 22–25-month-old mice) (Shah et al., 2006). These reports show that basal IL-1β expression is significantly higher in the hippocampus in aged mice than in young mice; however, there has been no comparison between 3-month-old and 6-month-old mice. In contrast, IL-1β mRNA expression in the hippocampal dentate gyrus (DG) under basal conditions was higher and significantly increased after hippocampal tetanic stimulation in young rats (3 months old) compared with that in adult rats (12–16 months old) (Balschun et al., 2003). These findings suggest that physiological IL-1β levels are definitely high in the hippocampus in young animals and lower in middle-aged adult animals but then increase in aged animals.

Therefore, we hypothesized that hippocampal IL-1β upregulation has a beneficial effect via IL-1r1 on spatial learning and memory in young mice, but the effect is diminished in adult mice with a decrease in IL-1β levels. To confirm this hypothesis, we investigated the performance of 3-month-old and 6-month-old wild-type (wt) mice, IL-1β knockout mice (IL-1βko), and IL-1r1ko mice in the WM task and measured the concentration of IL-1β and IL-1α in the hippocampus and cortex using young and adult mice brain tissue.

## Materials and Methods

### Animals

The subjects were 3-month-old (young) and 6-month-old (adult) male IL-1βko mice (10 young and 8 adult mice; Tokyo University of Science, Noda, Chiba, Japan) and IL-1r1ko mice (Glaccum et al., 1997) (11 young and 10 adult mice; Jackson Laboratories, Bar Harbor, ME, United States), and corresponding age-matched wt controls were used (26 young and 19 adult mice; C57BL/6J). We used separate young mice and adult mice in each experiment. We needed age-matched control mice to pair with the knockout mice and be maintained under the same experimental conditions, including breeding, feeding, and handling, for each experiment. Wt littermates should be used as controls for the knockout mice; however, having at least five age-matched homozygous mice and wt mice for each group at the same time was fundamentally very difficult. Therefore, we produced the knockout mice by mating the homozygous mice, which were backcrossed seven to eight times to C57BL/6J mice, and we used C57BL/6J mice as wt controls. In addition, we carefully arranged the breeding conditions of the mice. Although 3-month-old mice are generally considered to be adults, we called the group of 3-month-old mice young mice, and the group of 6-month-old mice adult mice to distinguish the two groups in this study. IL-1r1ko mice do not express the IL-1 receptor type 1. All known biological functions of IL-1 are mediated by this receptor, and the IL-1r1ko mice show impaired responses to IL-1α and IL-1β (Labow et al., 1997). The mice were housed individually with the conditions, such as handling method and duration, maintained as consistent as possible in a room at 24 ± 2°C with a standard 12-h light:dark cycle, and mice had access to standard chow and water *ad libitum*. All study protocols were approved by the Animal Care and Use Committee of Tokyo Women’s Medical University (ethics numbers: AE16-51, GE16-42).

### Assessment of Spatial Memory Task Learning in the WM

The WM consisted of a 100-cm diameter circular pool filled with water (25 ± 1°C) mixed with white bath salts to make the water opaque. Animals were pre-trained on the task 1 day before the spatial memory experiment. During the pre-training session, the mice were required to locate and climb onto a visible platform (PF) (13 cm in diameter elevated 1 cm above the surface of the water) located in the center of the pool. The maximal trial duration was 15 s. Pre-training consisted of three trials over a 1-day period, with 10-s breaks between trials. The mice performed the spatial memory task the day after pre-training. During the spatial memory task, the PF was placed 0.5 cm below the surface of the water so that it was no longer visible to the mouse, and the submerged PF was placed in a fixed position. For each training trial, the mouse was placed in the tank at a random location and allowed to swim freely. Once the mouse found the PF and climbed onto it, it was allowed to remain on the PF for 10 s. If the mouse did not locate the PF after 60 s, the mouse was guided to the PF and allowed to remain on it for 10 s. The mice performed three trials per block, and we used the average of the three trials, with a 1-min inter-trial interval. Each mouse performed nine blocks of three training trials over 3 days (three blocks per day with a 1.5-h interval between blocks). The illuminated and distal visual cues on the walls and ceiling were kept the same throughout the experiment. We assessed the learning of the WM spatial memory task by measuring the latency to reach the PF across blocks or days. Moreover, we investigated the path length to the PF and the velocity to estimate the locomotor skill in mice.

Probe tests were conducted immediately after training (0 day) and at 1 day, 1 week, and 1 month after training. We omitted the day 0 test in the experiment using young IL-1r1ko mice with wt control mice to investigate the effect of the day 0 probe trial. During the probe test, the PF was removed from the pool, and each mouse was allowed to search freely for 60 s. Each mouse performed three trials, with a 10-s interval between trials. We measured the total duration the mouse spent searching the target quadrant. The test lasted 60 s; therefore, the expected duration spent in the target quadrant was 15 s. We compared the observed total search duration to this chance level. We calculated the ratio of the duration spent in the target quadrant against the chance level as an analysis of the probe test to investigate the effect of IL-1 in the memory extinction process. A video camera above the pool was connected to a computerized tracking system that monitored and automatically measured the latency and the path length for each mouse to reach the PF during the training trials. During the probe test, the total duration the mouse spent searching the target quadrant was monitored and measured. The video data were analyzed by using Top Scan software (Clever Sys., Inc., Reston, VA, United States).

### Hippocampal Cytokines

Mice were subjected to three blocks of three training trials, and the next day, they were decapitated to obtain the cytokine data associated with the WM 18-h data. We used six young mice and seven adult mice that did not participate in the WM task and six young mice and seven adult mice that were trained on the WM task 18 h prior (WM 18-h condition). After the animals were deeply anesthetized and decapitated, the bilateral hippocampi and cortexes were removed and homogenized in ice-cold lysis buffer containing 25 mM HEPES, pH 7.43, 0.1% [(3-cholamidopropyl)dimethyl-ammonio]1-propanesulfonate, 5 mM MgCl_2_, 1.3 mM EDTA, 1 mM EGTA, 10 mg/ml pepstatin, aprotinin, and leupeptin, and 1 mM PMSF. The homogenates were centrifuged (15 min at 50,000 × *g*) and stored at -80°C. We measured the concentrations of hippocampal IL-1β and IL-1α by using homogeneous time-resolved fluorescence (HTRF) with mouse IL-1β (63ADK010PEB-JP) and mouse IL-1α (63ADK068PEB-JP) cytokine determination kits from Cisbio Japan (Tokyo, Japan). For the protein assay, we used a Coomassie (Bradford) Protein Assay Kit from Thermo Scientific (Rockford, IL, United States).

### Statistical Analysis

Data are presented as the average ± standard error (*SE*). Statistical analyses were performed using Student’s *t*-test and repeated measures ANOVA. SAS version 9.4 (SAS Institute, Cary, NC, United States) was used for the statistical analyses. Significance was determined at a *p*-value < 0.05.

## Results

### Spatial Memory Task Learning in the WM in Young IL-1βko and IL-1r1ko Mice

**FIGURE 1:**
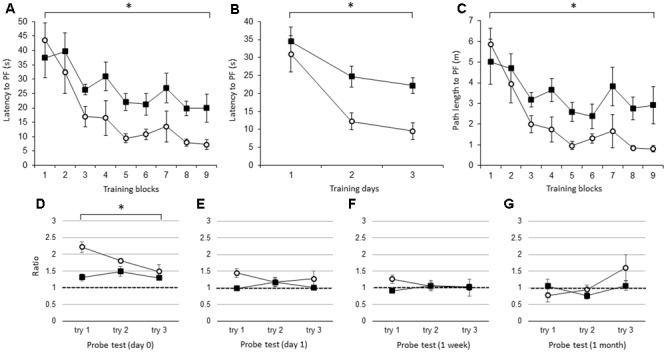
The effect of IL-1β during the WM spatial memory task in young mice. **(A)** Latency to reach the hidden platform (PF) during the training period in young IL-1β knockout mice (IL-1βko) mice (closed square, *n* = 7) and age-matched wt mice (open circle, *n* = 7) showing the average of the three trials in each block. **(B)** Latency to reach the hidden PF during the training period in young IL-1βko mice (closed square, *n* = 7) and age-matched wt mice (open circle, *n* = 7) showing the average of the nine trials on each day. **(C)** Path length to reach the hidden PF during the training period in young IL-1βko mice (closed square, *n* = 7) and age-matched wt mice (open circle, *n* = 7) showing the average of the three trials in each block. **(D–G)** The ratio of the total duration spent in the target quadrant in the probe trials in young IL-1βko mice (closed square, *n* = 7) and wt mice (open circle, *n* = 7) at 0 day **(D)**, 1 day **(E)**, 1 week **(F)**, and 1 month **(G)** after the final training trial. ^∗^*p* < 0.01.

Beginning with the average of the three trials per block, the latency to the PF was significantly longer in the IL-1βko mice (*N* = 7) than in the wt mice (*N* = 7) [**Figure 1A**; *F*(1,12) = 9.42, *p* = 0.0097], and there was no interaction. There was also an effect of time [*F*(8,96) = 10.52, *p* < 0.0001]. In **Figure 1B**, we compared the latency to the PF in the IL-1βko and wt mice using the average of the nine trials per day [**Figure 1B**; *F*(1,12) = 9.42, *p* = 0.0097] and showed an effect of time [*F*(2,24) = 17.45, *p* < 0.0001]. The path length to reach the PF was also significantly longer in the IL-1βko mice (*N* = 7) than in the wt mice (*N* = 7) [**Figure 1C**; *F*(1,12) = 9.56, *p* = 0.0093], and there was no interaction. There was also an effect of time [*F*(8,96) = 9.14, *p* < 0.0001]. Moreover, there was no significant difference in the velocity between IL-1βko and wt mice [*F*(1,12) = 0.43, *p* = 0.5266] with no effect of time [*F*(8,96) = 1.62, *p* = 0.1287], suggesting that the locomotor skills of the IL-1βko mice and wt mice did not differ. Thus, young IL-1β ko mice exhibited impaired learning in the WM spatial memory task. In the last trial of the 9th block, the average latency to reach the PF was 7.2 ± 1.7 s in the wt group and 19.9 ± 4.9 s in the IL-1βko group, which was over twice the latency observed in the wt group. In the probe tests, we compared the ratio of the total duration spent in the target quadrant against chance between young wt and IL-1βko mice each day. There was a significant difference in the probe test on day 0 [**Figure 1D**; *F*(1,12) = 10.64, *p* = 0.0068]. In contrast, there were no significant differences on day 1 [**Figure 1E**; *F*(1,12) = 3.15, *p* = 0.1012] or at 1 week [**Figure 1F**; *F*(1,11) = 2.57, *p* = 0.1370] or 1 month [**Figure 1G**; *F*(1,12) = 0.51, *p* = 0.4883] after training. The significant difference on day 0 may not be indicative of memory extinction because the low ratio in the first trial of the probe test was affected by the low memory consolidation shown in the last trial of training, and the ratio dropped to chance level in the probe test on day 1 in the IL-1βko mice.

**FIGURE 2:**
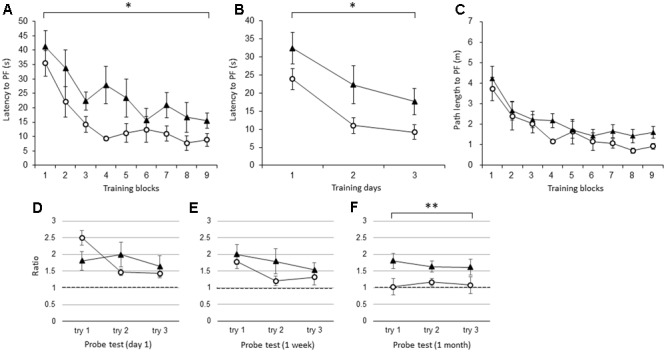
The effect of IL-1r1 during the WM spatial memory task in young mice. **(A)** Latency to reach the hidden PF during the training period in young IL-1r1ko mice (closed triangle, *n* = 7) and age-matched wt mice (open circle, *n* = 7) showing the average of the three trials in each block. **(B)** Latency to reach the hidden PF during the training period in young IL-1r1ko mice (closed triangle, *n* = 7) and age-matched wt mice (open circle, *n* = 7) showing the average of the nine trials on each day. **(C)** Path length to reach the hidden PF during the training period in young IL-1r1ko mice (closed triangle, *n* = 7) and age-matched wt mice (open circle, *n* = 7) showing the average of the three trials in each block. **(D–F)** The ratio of the total duration spent in the target quadrant in the probe trials in young IL-1r1ko mice (closed triangle, *n* = 7) and wt mice (open circle, *n* = 7) at 1 day **(D)**, 1 week **(E)**, and 1 month **(F)** after the final training trial. ^∗^*p* < 0.05, ^∗∗^*p* < 0.01.

Like the young IL-1βko mice, the young IL-1r1ko mice showed an impairment in learning in the WM spatial memory task. There was a significant difference in the latency to the PF between the young IL-1r1ko mice (*N* = 7) and the wt mice (*N* = 7) when the average of the three trials in each block was compared [**Figure 2A**; *F*(1,12) = 5.34, *p* = 0.0394] with no interaction. There was also an effect of time [*F*(8,96) = 11.91, *p* < 0.0001]. There was also a difference in the latency across training days when the average of the nine trials each day was compared [**Figure 2B**; *F*(1,12) = 5.34, *p* = 0.0394] with an effect of time [*F*(2,24) = 14.16, *p* < 0.0001]. There was no significant difference in the path length to reach the PF between the IL-1r1ko mice (*N* = 7) and the wt mice (*N* = 7) [**Figure 2C**; *F*(1,12) = 2.79, *p* = 0.1204] with an effect of time [*F*(8,96) = 11.73, *p* < 0.0001] due to the slower velocity of the IL-1r1ko mice; however, there was no significant difference in the velocity between the IL-1r1ko and wt mice [*F*(1,12) = 4.46, *p* = 0.0562]. The results suggest that the locomotor skill was slightly but not significantly inferior in the IL-1r1ko mice. Thus, young IL-1r1ko mice also revealed impaired learning of the spatial memory task in the WM. In the probe tests, we compared the ratio of the total duration spent in the target quadrant between young wt and IL-1r1ko mice each day. There were no significant differences in the probe test on day 1 [**Figure 2D**; *F*(1,10) = 0.00, *p* = 0.9862] or 1 week after training [**Figure 2E**; *F*(1,12) = 1.52, *p* = 0.2406], but there was a significant difference 1 month after training [**Figure 2F**; *F*(1,9) = 11.03, *p* = 0.0089] because the ratio of the duration in the target quadrant was maintained at high levels in the IL-1r1ko mice, which reveals that IL-1r1ko mice had an impairment in memory extinction.

### Spatial Memory Task Learning in the WM in Adult IL-1βko and IL-1r1ko Mice

**FIGURE 3:**
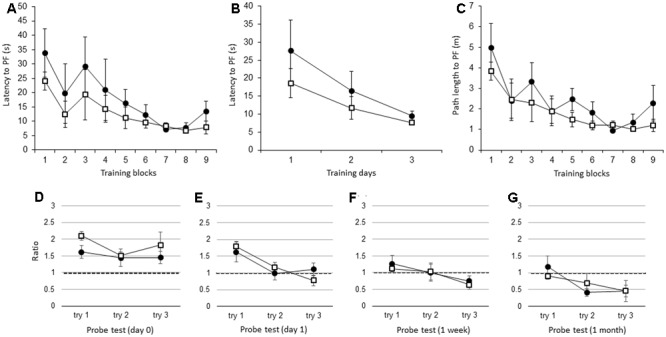
The effect of IL-1β during the WM spatial memory task in adult mice. **(A)** Latency to reach the hidden PF during the training period in adult IL-1βko mice (open square, *n* = 5) and age-matched wt mice (closed circle, *n* = 5) showing the average of the three trials in each block. **(B)** Latency to reach the hidden PF during the training period in adult IL-1βko mice (open square, *n* = 5) and age-matched wt mice (closed circle, *n* = 5) showing the average of the nine trials on each day. **(C)** Path length to reach the hidden PF during the training period in adult IL-1βko mice (open square, *n* = 5) and age-matched wt mice (closed circle, *n* = 5) showing the average of the three trials in each block. **(D–G)** The ratio of the total duration spent in the target quadrant in the probe trials in adult IL-1βko mice (open square, *n* = 5) and wt mice (closed circle, *n* = 5) at 0 day **(D)**, 1 day **(E)**, 1 week **(F)**, and 1 month **(G)** after the final training trial.

There were no significant differences in the latency to the PF between adult IL-1βko mice (*N* = 5) and wt mice (*N* = 5) when the average of the three trials in each block was compared [**Figure 3A**; *F*(1,8) = 1.26, *p* = 0.2938], but there was an effect of time [*F*(8,64) = 4.25, *p* = 0.0004]. There was also no difference in the average of the nine trials per day [**Figure 3B**; *F*(1,8) = 1.26, *p* = 0.2938], but there was an effect of time [*F*(2,16) = 6.44, *p* = 0.0089]. There was no significant difference in the path length to reach the PF between the IL-1βko mice and the wt mice [**Figure 3C**; *F*(1,8) = 2.57, *p* = 0.1475], but there was an effect of time [*F*(8,64) = 4.96, *p* < 0.0001]. Additionally, there was no significant difference in the velocity between the IL-1βko mice and the wt mice [*F*(1,8) = 0.02, *p* = 0.8807]. During the probe test, there was no significant difference in the ratio of the total duration spent in the target quadrant between adult wt and IL-1βko mice on day 0 [**Figure 3D**; *F*(1,7) = 2.27, *p* = 0.1758] and day 1 [**Figure 3E**; *F*(1,8) = 0.00, *p* = 0.9855] as well as 1 week [**Figure 3F**; *F*(1,6) = 0.35, *p* = 0.5733] and 1 month [**Figure 3G**; *F*(1,7) = 0.00, *p* = 0.9868] after training.

**FIGURE 4:**
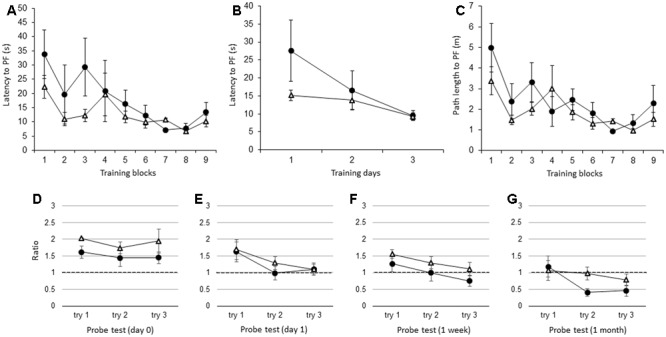
The effect of IL-1r1 during the WM spatial memory task in adult mice. **(A)** Latency to reach the hidden PF during the training period in adult IL-1r1ko mice (open triangle, *n* = 5) and age-matched wt mice (closed circle, *n* = 5) showing the average of the three trials in each block. **(B)** Latency to reach the hidden PF during the training period in adult IL-1r1ko mice (open triangle, *n* = 5) and age-matched wt mice (closed circle, *n* = 5) showing the average of the nine trials on each day. **(C)** Path length to reach the hidden PF during the training period in adult IL-1r1ko mice (open triangle, *n* = 5) and age-matched wt mice (closed circle, *n* = 5) showing the average of the three trials in each block. **(D–G)** The ratio of the total duration spent in the target quadrant in the probe trials in adult IL-1r1ko mice (open triangle, *n* = 5) and wt mice (closed circle, *n* = 5) at 0 day **(D)**, 1 day **(E)**, 1 week **(F)**, and 1 month **(G)** after the final training trial.

Similar to the adult IL-1βko mice, there were no significant differences in the latency to the PF between the adult IL-1r1ko (*N* = 5) and the wt (*N* = 5) mice when the average of the three trials in each block was compared [**Figure 4A**; *F*(1,8) = 1.34, *p* = 0.2799], but there was an effect of time [*F*(8,64) = 3.80, *p* = 0.0011]. **Figure 4B** shows that there was no difference between the two groups in the average of the nine trials per day [**Figure 4B**; *F*(1,8) = 1.34, *p* = 0.2799], but there was an effect of time [*F*(2,16) = 5.13, *p* = 0.0190]. There was no significant difference in the path length to reach the PF between the IL-1r1ko mice and the wt mice [**Figure 4C**; *F*(1,8) = 3.10, *p* = 0.1162], but there was an effect of time [*F*(8,64) = 4.18, *p* = 0.0005]. Additionally, there was no significant difference in the velocity between the IL-1r1ko mice and the wt mice [*F*(1,8) = 0.01, *p* = 0.9144]. During the probe test, there was no significant difference in the ratio of the total duration spent in the target quadrant between the adult wt and IL-1r1ko mice on day 0 [**Figure 4D**; *F*(1,6) = 3.74, *p* = 0.1012] and day 1 [**Figure 4E**; *F*(1,7) = 0.14, *p* = 0.7199] as well as 1 week [**Figure 4F**; *F*(1,7) = 0.84, *p* = 0.3912] and 1 month [**Figure 4G**; *F*(1,6) = 3.78, *p* = 0.0999] after training.

### The Basal Concentrations of IL-1β and IL-1α in the Hippocampus and Cortex

**FIGURE 5:**
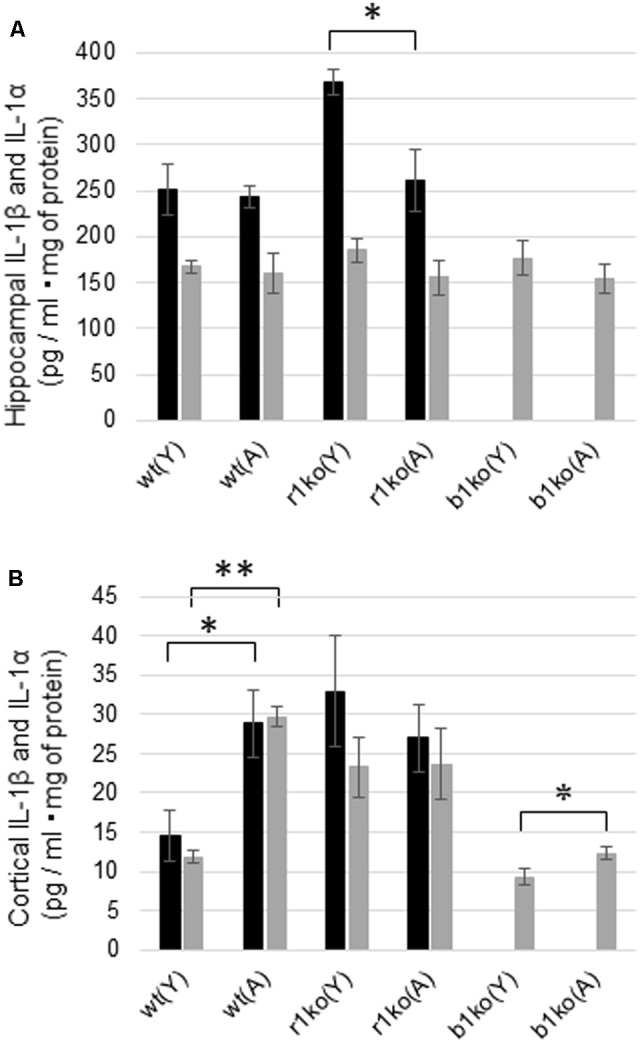
Basal hippocampal and cortical concentrations of IL-1α and IL-1β in young and adult wt mice, IL-1r1ko mice, and IL-1βko mice. **(A)** Concentrations of basal levels of hippocampal IL-1β (black bar) and IL-1α (gray bar). **(B)** Concentrations of basal levels of cortical IL-1β (black bar) and IL-1α (gray bar). wt, wild-type mice; r1ko, IL-1r1ko mice; b1ko, IL-1βko mice; (Y), young; (A), adult. ^∗^*p* < 0.05, ^∗∗^*p* < 0.0001.

The hippocampal concentration of basal IL-1α was not significantly different between the young (*N* = 6) and adult (*N* = 7) wt mice (**Figure 5A**, *p* = 0.3944, *t* = 0.274) or between the young and adult IL-1βko mice (*N* = 3, **Figure 5A**, *p* = 0.2085, *t* = 0.939). In addition, there was also no significant difference in the basal levels of hippocampal IL-1β between the young (*N* = 6) and adult (*N* = 7) wt mice (**Figure 5A**, *p* = 0.4035, *t* = 0.250). The hippocampal concentration of IL-1β was high in young IL-1r1ko mice (*N* = 4) compared with adult IL-1r1ko mice (*N* = 5) (**Figure 5A**, *p* = 0.01644, *t* = 2.6513). Interestingly, basal levels of IL-1α and IL-1β in the cortex were significantly higher in the adult wt mice (*N* = 6) than in the young wt mice (*N* = 6) (**Figure 5B**, *p* < 0.0001, *t* = 11.77; **Figure 5B**, *p* = 0.0115, *t* = 2.68). Moreover, cortical IL-1α levels were significantly higher in adult IL-1βko mice than in young IL-1βko mice (*N* = 3, **Figure 5B**, *p* = 0.02875, *t* = 2.27).

### Hippocampal Concentrations of IL-1β and IL-1α 18 h after the WM Task

**FIGURE 6:**
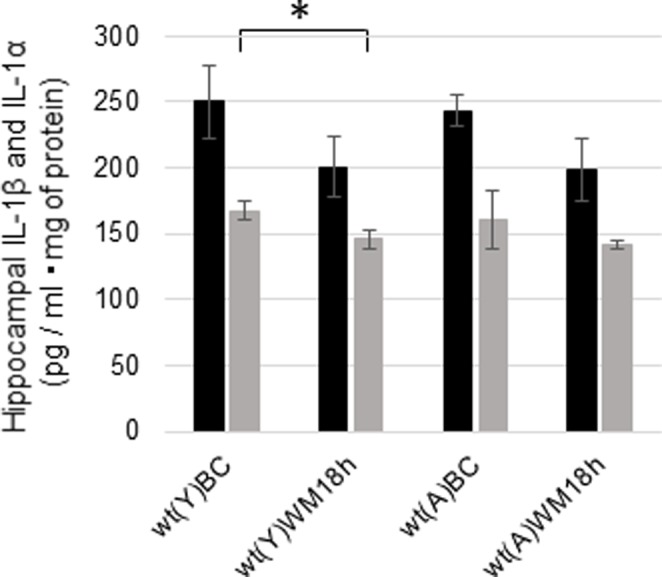
Changes in the hippocampal concentrations of IL-1β and IL-1α in young and adult wt mice after the WM task. Concentrations of hippocampal IL-1β (black bar) and IL-1α (gray bar) under basal conditions (BC) and 18 h after the WM task (WM 18 h). wt, wild-type mice; (Y), young; (A), adult. ^∗^*p* < 0.05.

We measured the concentrations of hippocampal IL-1β and IL-1α 18 h after the WM task in young and adult wt mice to investigate whether IL-1 signaling changed after the WM task. The concentrations of IL-1β decreased after the WM task but not significantly in young (**Figure 6**; *p* = 0.0980, *t* = 1.39, *N* = 6 in each group) and adult mice (**Figure 6**; *p* = 0.0594, *t* = 1.68, *N* = 7 in each group). Additionally, IL-1α was significantly decreased in young mice after the WM task (**Figure 6**; *p* = 0.0266, *t* = 2.19, *N* = 6 in each group), but no difference was observed in adults (**Figure 6**; *p* = 0.2076, *t* = 0.844, *N* = 7 in each group).

## Discussion

In this study, we hypothesized that physiological IL-1β levels and their effects vary with age in the brain. Our hypothesis is that IL-1β has a beneficial effect via IL-1r1 on hippocampal spatial memory in young mice, whereas the effect is diminished in adult mice. Therefore, we expected that there would be a significant impairment in learning a WM spatial memory task in young IL-1βko mice and IL-1r1ko mice but not in adult IL-1βko mice and IL-1r1ko mice. In addition, we studied whether physiological IL-1 levels were high in the basal hippocampus and further increased 18 h after WM test in young wt mice compared to that in adult mice.

The results show that young IL-1β ko and IL-1r1ko mice showed an impairment in spatial memory task learning in the WM, suggesting that brain IL-1β activates IL-1r1 to facilitate hippocampal spatial learning and memory in young mice (**Figures 1A,B, 2A,B**). Because the IL-1α and IL-1β genes have been reported to be highly expressed in association with memory recall (Scholz et al., 2016), IL-1 might play an important role in hippocampal spatial memory in young mice. Furthermore, there was no significant difference in the velocity between IL-1β and wt mice or between IL-1r1ko and wt mice, suggesting that locomotor behavior was not impaired in either IL-1βko or IL-1r1ko mice. Thus, young IL-1βko mice and IL-1r1ko mice specifically exhibited an impairment in spatial memory task learning in the WM. Therefore, brain IL-1 facilitates the learning of the spatial memory task in the WM in young mice.

In probe trials, we estimated the memory extinction process by examining the gradual decline in the ratio of the duration of time spent in the target quadrant. We omitted the day-0 WM probe test in the experiment using the young IL-1r1ko mice; therefore, the decline in the ratio was delayed until 1 week after training in the wt mice (**Figure 2E**). However, the ratio was close to chance level in the WM probe test 1 month after training (**Figure 2F**). In contrast, the ratio was maintained at a high level 1 month after training in the IL-1r1ko mice (**Figure 2F**), suggesting that the memory extinction process is impaired in IL-1r1ko mice. This extended memory was not observed in IL-1βko mice (**Figure 1G**); therefore, IL-1α might be related to the spatial memory extinction process. Because IL-1α mRNA is known to be expressed after learning training (Depino et al., 2004), inducible IL-1α might be related to memory extinction. Moreover, IL-1α expression is further increased under physiological temperatures (34–36°C) (Ross et al., 2003); therefore, IL-1α might be increased in the brain after the training trials. Furthermore, clinical research has shown that genetic variants in IL-1 genes are involved in lower cognition associated with inflammation (Benke et al., 2011). Therefore, IL-1α plays a role in cognitive function.

Unlike in the young IL-1βko and IL-1r1ko mice, spatial memory task learning in the WM was not impaired in the adult IL-1βko and IL-1r1ko mice relative to the wt mice (**Figures 3A,B, 4A,B**). Moreover, the impairment in the memory extinction task in the 1-month WM probe test found in young IL-1r1ko mice was diminished in the adult IL-1r1ko mice (**Figure 4G**). Our current results suggest that IL-1β and IL-1α have a beneficial effect on hippocampal spatial learning and memory that is limited to young mice.

Unexpectedly, there were no significant differences in the basal concentrations of hippocampal IL-1β and IL-1α between young and adult wt mice (**Figure 5A**). In addition, hippocampal IL-1β and IL-1α did not increase 18 h after WM in young or adult mice in our study (**Figure 6**). As IL-1β mRNA was increased during LTP in the DG just 8 h after tetanic stimulation in 3-month-old young rats (Balschun et al., 2003), we need to investigate the concentration of IL-1β and IL-1α in the hippocampus at an earlier timepoint. It is unclear whether IL-1β in hippocampal neurons locally facilitates learning of the hippocampal WM spatial memory task in young mice. In addition, IL-1α and IL-1β levels were significantly higher in the cortex in adult wt mice than in young wt mice (**Figure 5B**). The RSC plays an integral role in spatial memory (Czajkowski et al., 2014; Miller et al., 2014; Todd and Bucci, 2015). In addition, several lines of evidence indicate that the prefrontal cortex is reported to have a crucial function in spatial memory (Euston et al., 2012; Sapiurka et al., 2016), in particular in spatial memory extinction (Mendez-Couz et al., 2014). Therefore, cortical IL-1 may play a role in hippocampal spatial learning and memory. We also need to investigate the role of cortical IL-1 in the future.

We demonstrated an age-related change in IL-1β function. Viviani and Boraso (2011) suggest that IL-1β plays a role in synaptic plasticity and contributes to the functional deficit that characterizes the aged brain. For example, IL-1β increases GABAergic inhibition to impair LTP (Hellstrom et al., 2005). If these functions increase with age, the beneficial effect of IL-1β to facilitate memory in young mice might disappear. Another researcher has suggested a potential role for neuroinflammation in the aged brain (Sparkman and Johnson, 2008). The aging brain shows a shift in the homeostatic balance of inflammatory mediators to a proinflammatory state. In addition, inflammation impairs synaptic plasticity and cognition by activating one or more members of the mitogen activated protein (MAP) kinase family, such as p38 MAP kinase. Neuroinflammatory mediators also modulate neuronal Ca^2+^ signaling and homeostasis, which affect synapses (Sama and Norris, 2013). Sensitivity to IL-1β might be augmented in aged hippocampal synapses, and the increased synaptic sensitivity could be due to the reconfiguration of the IL-1 receptor subunit (Prieto et al., 2015).

Finally, we investigated the age-related production of hippocampal IL-1β and the effect of the WM task as a physiological stressor. A previous study showed that basal IL-1β levels in the hippocampus are higher in 22- to 25-month-old mice than in 5- to 6-month-old mice (Shah et al., 2006). In addition, peripheral stimulation with LPS also increases IL-1β levels in the hippocampus in 22- to 24-month-old mice more than in 3- to 6-month-old mice (Bilbo et al., 2008; Richwine et al., 2008). These findings suggest that the basal IL-1β levels may be high in old mice and may increase further after physiological stress such as neuroinflammation. We also measured the concentrations of hippocampal IL-1β and IL-1α 18 h after the WM task in young and adult wt mice to investigate whether basal levels of IL-1 were increased after the WM task. Our data show that the hippocampal IL-1 levels were decreased 18 h after the WM task in adult and young mice (**Figure 6**). Previous reports have shown that exposure to a forced swim test decreases the concentration of IL-1 in the cortex and hippocampus (Deak et al., 2003) and that running decreases the levels of IL-1β in the hippocampus (Chennaoui et al., 2008). Therefore, the brain condition 18 h after the WM task might be similar to that after a swimming exercise, which decreases IL-1β.

In this study, we observed a beneficial effect of IL-1 on spatial memory task learning in the WM in young mice, and the effect was diminished in adult mice. In conclusion, brain IL-1 regulates learning in a WM spatial memory task in young mice.

## Author Contributions

TT, KF, KY, YI, and MK made substantial contribution to the design, acquisition, analysis, and interpretation of the experimental work, and TT wrote the manuscript.

## Conflict of Interest Statement

The authors declare that the research was conducted in the absence of any commercial or financial relationships that could be construed as a potential conflict of interest.
